# Replication and Virus-Induced Transcriptome of HAdV-5 in Normal Host Cells versus Cancer Cells - Differences of Relevance for Adenoviral Oncolysis

**DOI:** 10.1371/journal.pone.0027934

**Published:** 2011-11-30

**Authors:** Dominik E. Dorer, Frank Holtrup, Kurt Fellenberg, Johanna K. Kaufmann, Sarah Engelhardt, Jörg D. Hoheisel, Dirk M. Nettelbeck

**Affiliations:** 1 Helmholtz-University Group Oncolytic Adenoviruses, German Cancer Research Center (Deutsches Krebsforschungszentrum [DKFZ]), Department of Dermatology, Heidelberg University Hospital, Heidelberg, Germany; 2 Division of Functional Genome Analysis, German Cancer Research Center (Deutsches Krebsforschungszentrum [DKFZ]), Heidelberg, Germany; 3 Department of Plant Physiology, Ruhr University Bochum, Bochum, Germany; The University of Chicago, United States of America

## Abstract

Adenoviruses (Ads), especially HAdV-5, have been genetically equipped with tumor-restricted replication potential to enable applications in oncolytic cancer therapy. Such oncolytic adenoviruses have been well tolerated in cancer patients, but their anti-tumor efficacy needs to be enhanced. In this regard, it should be considered that cancer cells, dependent on their tissue of origin, can differ substantially from the normal host cells to which Ads are adapted by complex virus-host interactions. Consequently, viral replication efficiency, a key determinant of oncolytic activity, might be suboptimal in cancer cells. Therefore, we have analyzed both the replication kinetics of HAdV-5 and the virus-induced transcriptome in human bronchial epithelial cells (HBEC) in comparison to cancer cells. This is the first report on genome-wide expression profiling of Ads in their native host cells. We found that E1A expression and onset of viral genome replication are most rapid in HBEC and considerably delayed in melanoma cells. In squamous cell lung carcinoma cells, we observed intermediate HAdV-5 replication kinetics. Infectious particle production, viral spread and lytic activity of HAdV-5 were attenuated in melanoma cells versus HBEC. Expression profiling at the onset of viral genome replication revealed that HAdV-5 induced the strongest changes in the cellular transcriptome in HBEC, followed by lung cancer and melanoma cells. We identified prominent regulation of genes involved in cell cycle and DNA metabolism, replication and packaging in HBEC, which is in accord with the necessity to induce S phase for viral replication. Strikingly, in melanoma cells HAdV-5 triggered opposing regulation of said genes and, in contrast to lung cancer cells, no weak S phase induction was detected when using the E2F promoter as reporter. Our results provide a rationale for improving oncolytic adenoviruses either by adaptation of viral infection to target tumor cells or by modulating tumor cell functions to better support viral replication.

## Introduction

Adenoviruses (Ads) are emerging cancer therapeutics based on their potency to infect and lyse cancer cells, a process termed viral oncolysis [1], [2]. This regimen features a unique amplification effect as infected tumor cells produce progeny viruses that spread infection in the tumor. A further advantage is that the mode of action of oncolytic Ads differs from conventional therapies, to which cancer cells frequently develop resistance. Restriction of virus replication to tumor cells is essentially required for the application of Ads in cancer therapy. In this regard, the extensive knowledge of Ad structure, genome organization and replication cycle combined with technologies for Ad engineering facilitates the rational development of oncolytic Ads [2], [3]. Indeed, oncolytic viruses with outstanding tumor selectivity have been engineered based on the closely related HAdV-2 and HAdV-5. This was achieved either by mutating gene functions that are complemented in cancer cells, but not in normal cells, or by targeting the expression of essential viral genes to tumor cells [1], [2], [4]. Several clinical trials have demonstrated that such engineered Ads are well tolerated in patients, but that their therapeutic potency needs improvement [5], [6]. In this context, the opportunity for rational engineering of Ads is again a key advantage as it facilitates the development of advanced oncolytic agents. Correspondingly, studies to improve Ad entry into cancer cells or to insert therapeutic genes into oncolytic Ads have been reported [2], [7], [8].

Adenoviral oncolysis necessitates efficient Ad replication in targeted cancer cells. Previous work in the field has not adequately considered that cancer cells, dependent on the tissue of origin, can differ substantially from normal Ad host cells. Thus, the virus does not come across the cellular environment it is adapted to by comprehensive virus-host cell interactions. In consequence, Ad replication, cell lysis and spread might be suboptimal. Specifically, HAdV-2 and -5 are evolutionary adapted to replicate in epithelial cells of the respiratory tract [9] but are being developed for therapy of a wide variety of tumor targets. Indeed, mutations of HAdV-5 that increase virus replication and spread in tumor cells have been reported [10]–[12]. One example is the deletion of *E1B19K*, which has anti-apoptotic activity. Deletion of *E1B19K* has resulted in strongly increased HAdV-5 replication and oncolysis in lung cancer cells. However, reduced replication has been reported in cancer cells derived from other tissues including melanoma cells [11], [13]–[15]. These observations again point at cell-type dependence of Ad-host cell interactions and, consequently, Ad replication efficiency: Differences in the apoptosis programming between normal and cancer cells, but also between different cancer cells most likely cause the different permissivity to *E1B19K*-deleted HAdV-5.

As obligatory intracellular parasites Ads are dependent on the cellular energy production and biosynthetic machinery. In fact, Ads have implemented diverse and intricate molecular mechanisms to establish conditions in the host cell that ensure efficient virus reproduction. These are best studied for HAdV-2 and -5 ([16], [17] and references therein). Correspondingly, products of early viral gene expression - besides providing proteins required for replication of the viral genome - manipulate the cellular environment to support virus replication by multiple mechanisms, especially by direct and indirect modulation of cellular transcription ([17]–[19] and references therein). The first viral gene expressed during Ad infection is E1A, which encodes a family of proteins resulting from alternative splicing. E1A proteins induce the expression of further early Ad genes and manipulate transcription of cellular genes directly or indirectly [20], [21]. The largest E1A protein (13S) contains a potent transactivation domain that induces transcription when E1A interacts with DNA-binding proteins. Both 13S and 12S E1A proteins bind to pRb, resulting in the release of E2F transcription factors from pRb-E2F complexes. The E2F proteins then activate transcription of viral and cellular genes via E2F-binding sites ([17], [19], [22], [23] and references therein). E2Fs widely induce cellular genes involved in the S phase of the cell cycle, which are also required for Ad genome replication. Further transcriptional activities of E1A proteins are mediated by interaction with a panel of cellular factors which inhibit or activate transcription, for example histone acetylases ([17] and references therein). Another Ad early protein, E1B55K, contains a strong transcription repression domain. By binding to the p53 transactivation domain it functionally switches p53 from a transcription activator to a repressor [24]. Furthermore, E1B55K together with E4ORF6 trigger degradation of p53 [25]. The resulting shut-off of p53-responsive pro-apoptotic genes counteracts the induction of apoptosis triggered by abnormal stimulation of the cell cycle by E1A.

The outstanding knowledge of Ad infection has been gained by extensive studies that were mostly performed with HAdV-2 or -5 in HeLa and other cancer cells and focused on individual Ad genes [17]. Thus, Ad infection in its normal environment of native host cells, e.g. primary epithelial cells of the respiratory tract for HAdV-5, has been rarely studied (most of these studies analyzed the selectivity of oncolytic Ads, see discussion). Note that HeLa cells differ from the normal Ad host cells, as they are of cervical cancer origin and have been extensively passaged. Moreover, they contain human papilloma virus genes which affect cell functions important for Ad replication. Studies that investigate how individual Ad genes affect cellular functions do not consider the complex network of virus-host interactions during virus infections. In this regard, microarray technology represents a powerful tool for genome-wide monitoring of reprogrammed cellular gene expression during Ad infection. Indeed, previous microarray studies have revealed that HAdV-2 and -5 infections target multiple cellular pathways [26]–[30]. These studies have been performed with HeLa cells and primary fibroblasts. How Ad infections modulate genome-wide gene expression of their native host cells has not been investigated to date. Consequently, a comparative analysis of Ad-induced gene expression profiles of tumor cells versus native Ad host cells, which is of interest for the development of oncolytic Ads, is not available to date.

In this study we therefore explored how Ad infection of cancer cells differs from Ad infection of their normal host cells. To this end, we performed a comparative analysis of HAdV-5 replication kinetics and lytic activity in primary bronchial epithelial cells, lung squamous cell carcinoma cells and melanoma cells. We chose this set of cells, as they represent normal HAdV-5 host cells, cells of tumors derived from HAdV-5 host cells and tumor cells derived from an unrelated cell type, respectively. We subsequently performed genome-wide expression profiling of HAdV-5 infection in normal bronchial epithelial cells and tumor cells. Cell type-dependent differences in the HAdV-5-induced cellular transcriptomes were assessed by bioinformatic analysis.

## Results

### Lytic potency and replication efficiency of HAdV-5 in normal human bronchial epithelial cells and cancer cells

**Figure 1:**
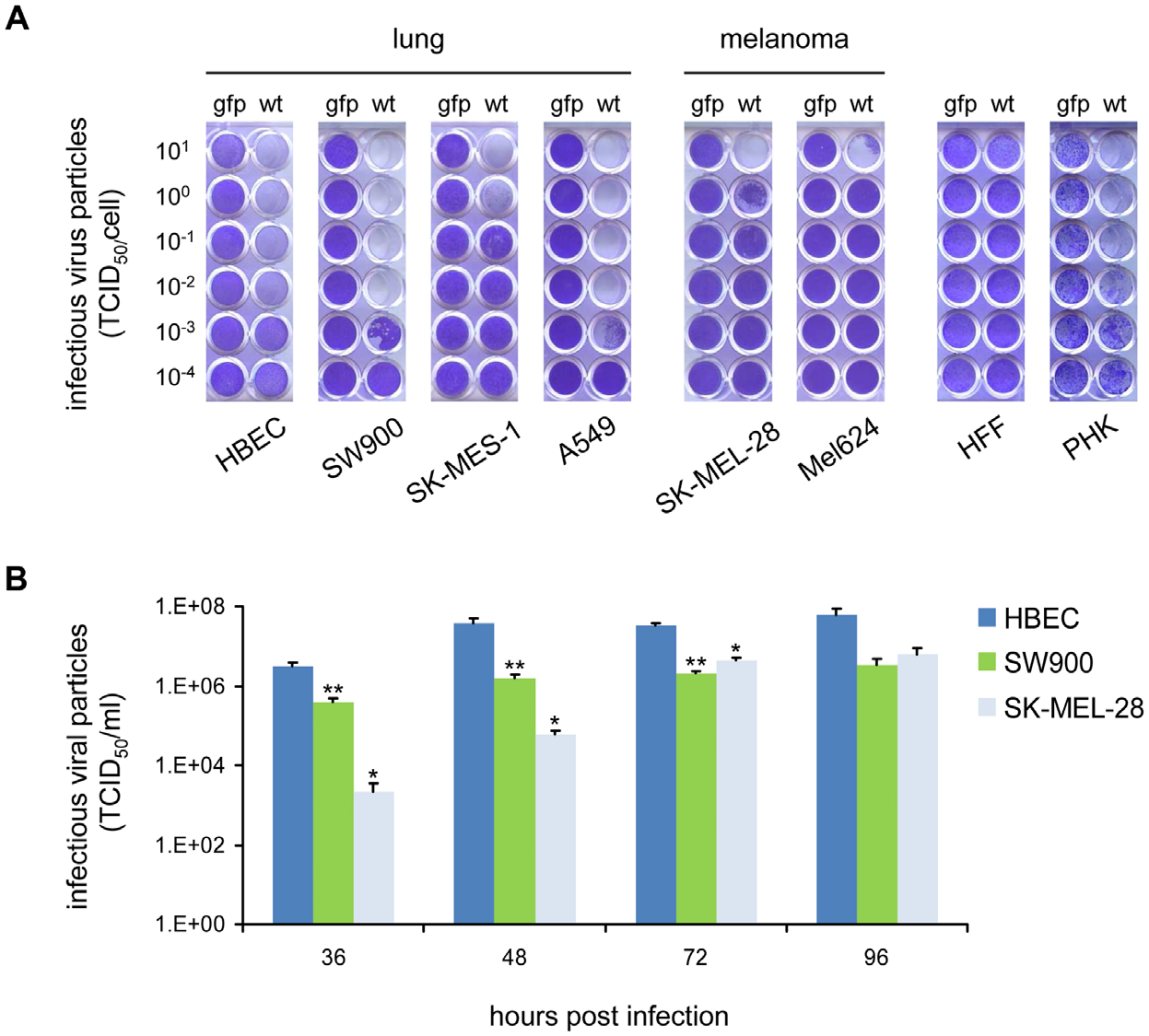
Comparison of HAdV-5 cytotoxicity and replication efficiency in primary cells and tumor cell lines. (A) Cytotoxicity: Various cell types were infected with either the wild type HAdV-5 (*wt*) or the replication-deficient control virus HAdV-5 CMV-gfp (*gfp*) at MOIs of 10^1^ to 10^−4^ TCID_50_/cell. Cells were primary human bronchial epithelial cells (HBEC, cells from one of two donors giving similar results are shown), squamous cell carcinoma of the lung (SK-MES-1, SW900), lung adenocarcinoma (A549), melanoma (SK-MEL-28, Mel624) primary human foreskin fibroblasts (HFF) and primary human keratinocytes (PHK). After incubation for eight days, surviving cells were fixed and stained with crystal violet. Lung cells (*left panels*) showed overall stronger cytotoxicity compared to melanoma cells (*middle panels*). (B) Replication: HBEC, SW900 or SK-MEL-28 cells were infected with 1 TCID_50_/cell of HAdV-5. After one hour incubation, inoculums were removed and cells were washed three times. Cells and supernatants were harvested at indicated time points and infectious virus particles were quantified by determination of TCID_50_. Bars represent mean values of triplicate infections and error bars standard deviations. Asterisks indicate statistical significance (*p≤0.05*) of differences between SK-MEL-28 and HBEC as well as SK-MEL-28 and SW900 (*), or between SW900 and HBEC (**). Increases of viral titers were significant (*p≤0.05*) for HBEC until 48 h and for SK-MEL-28 until 72 h post infection. For SW900 viral titers showed no significant increase after 36 h (*p = 0.056 for 48 h versus 36 h*).

We first assessed whether the efficiency of HAdV-5 spread-dependent cell lysis and replication differ between native HAdV-5 host cells and cancer cells. Primary human bronchial epithelial cells (HBECs) were used in our study as they represent the native HAdV-5 host cells of the respiratory epithelium most closely. They were compared to lung cancer cells SK-MES-1, SW900 (both squamous cell carcinoma), and A549 (adenocarcinoma), to melanoma cells SK-MEL-28 and Mel624, and to further human primary normal cells, namely fibroblasts and keratinocytes. In a cytotoxicity assay, we observed that spread-dependent cell lysis by HAdV-5 was similarly efficient in HBECs, SW900 and A549 cells, but attenuated in SK-MES-1 cells and even more in the two melanoma cell lines (Fig. 1A). Cytotoxicity of HAdV-5 for keratinocytes was similar to HBECs, whereas no cell killing was observed for primary fibroblasts at the time of measurement. These results clearly show that lytic potency of HAdV-5 is cell type-dependent and can be strongly reduced in cancer cells compared with HBECs. Of note, reduced lytic potency of HAdV-5 in SK-MEL-28 and Mel624 cells cannot be attributed to inefficient viral cell binding and entry, because these cells showed strong expression of the HAdV-5 receptor CAR (Fig. S1) and were even more susceptible to transduction by a replication-deficient HAdV-5 vector than HBECs, A549 and SW900 cells (Table S1). This is clear evidence that reduced lytic activity of HAdV-5 in melanoma cells is determined at a post-entry step of virus replication. In contrast, fibroblasts lacked CAR expression and were difficult to transduce. As cells were stained 8 days post-infection, which allows for several rounds of virus replication, the results of the cytotoxicity assay indicate differences in the efficacy of HAdV-5 replication and spread between melanoma cells and HBEC. Therefore, we next compared HAdV-5 replication in HBECs, SW900 and SK-MEL-28 more directly by quantification of infectious virus particle production during one round of replication (Fig. 1B). Infectious virus particle production by HBECs was >100-fold higher than for SK-MEL-28 at 36 h and 48 h post-infection. Virus titers peaked for HBEC at 48 h, whereas they continued to increase until 72 h for SK-MEL-28, when they peaked at a titer still lower than for HBECs. Infectious particle production by SW900 cells showed similar kinetics to HBECs, but remained approx 10-fold lower. We conclude that HAdV-5 replication is delayed in SK-MEL-28 cells compared with HBECs and SW900 cells, which is in accord with the cytotoxicity data.

### Kinetics of viral gene expression and genome replication of HAdV-5 in HBECs and cancer cells

**Figure 2:**
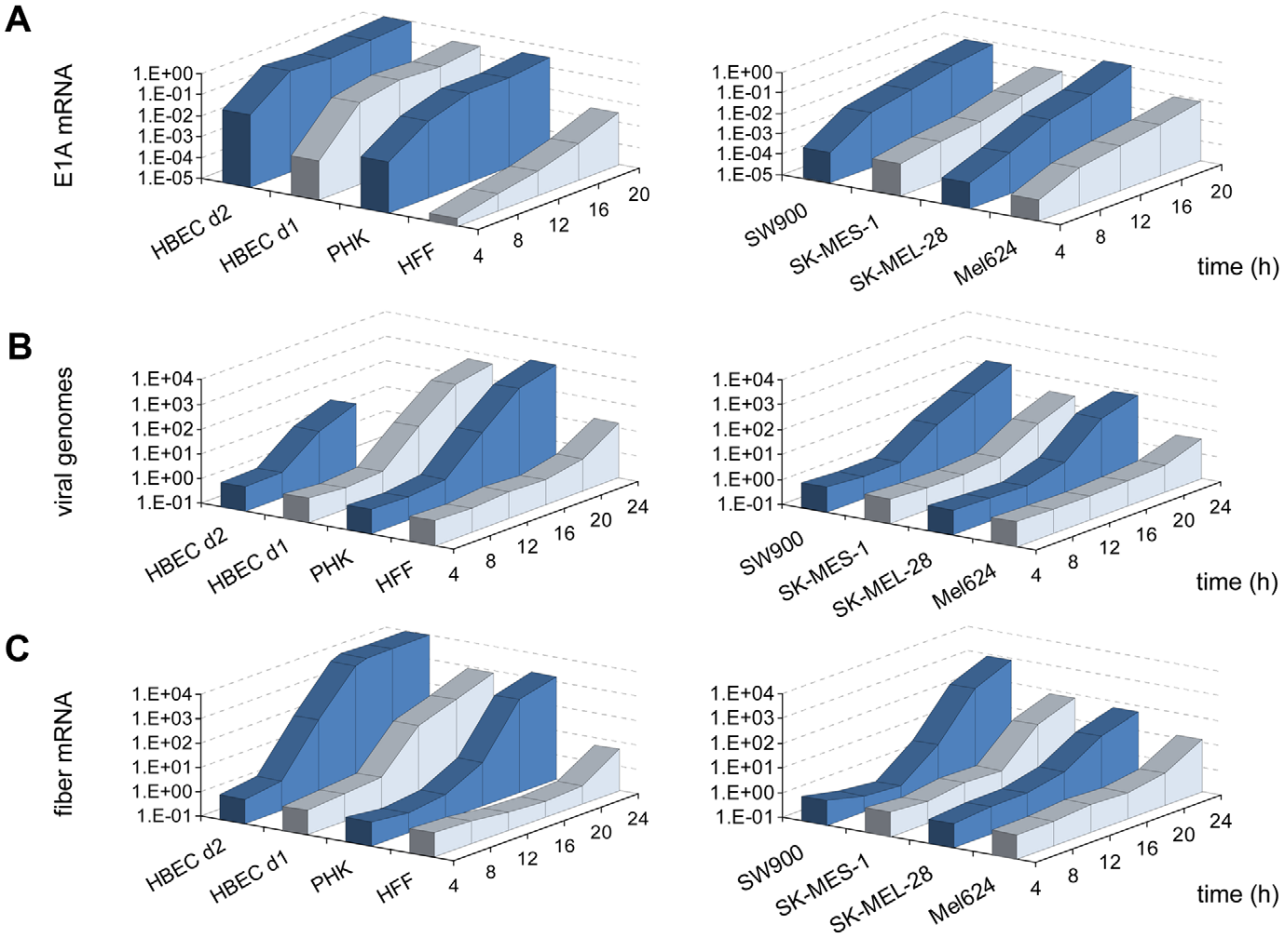
Viral gene expression and genome replication after HAdV-5 infection in primary cells and tumor cell lines. Human primary cells (*left panels*) and tumor cell lines (*right panels*) were infected with HAdV-5 at titers resulting in 80% infection efficiency (*see* *Fig. 1* *for names of cell types; HBEC d1, HBEC d2 are HBEC from different donors*). Inoculums were removed after one hour incubation. Total RNA and DNA was harvested for every indicated time point and was analyzed for E1A (A) and fiber (C) mRNA levels and for viral genome copies (B), respectively, by qPCR. Results of representative experiments are shown; repetition experiments yielded identical time points for the onset of E1A and fiber mRNA expression and virus genome replication. For HBEC d2, genome copy numbers were not determined at 20 h and 24 h, because of limited numbers of cells from this donor.

We next investigated the kinetics of HAdV-5 replication in HBECs and cancer cells in more detail by quantification of viral gene expression and genome replication (Fig. 2). As these experiments were used to define the time point of subsequent gene expression profiling, they were performed with the correspondingly standardized procedures, i.e. cells were cultured in microarray growth media and infected with virus titers resulting in 80% infection efficiency for each cell type (Table S1). Onset of viral DNA replication was delayed for melanoma cells (SK-MEL-28, 20 h; Mel624, 24 h) and SK-MES-1 cells (20 h) compared with SW900 cells (16 h) and HBEC (16 h or 12 h, dependent on the donor) (Fig. 2B). Primary fibroblasts showed a late (24 h), but primary keratinocytes an early (16 h) onset of viral DNA replication. These differences in HAdV-5 replication kinetics between the cell types correlated well with differences of viral lysis and infectious particle production (see Fig. 1). Analysis of E1A mRNA expression kinetics revealed that differences in onset of DNA replication between the cell types reflect corresponding differences in early gene expression: HBEC, SW900 and keratinocytes showed a rapid onset of E1A mRNA expression reaching near-maximum levels at 8 h post-infection. In contrast, for melanoma cells, SK-MES-1 and fibroblasts a more slowly and continuous increase in E1A mRNA expression was observed (Fig. 2A). The reason for the differences in E1A expression between cell lines is unclear. Transient transfection experiments with a reporter plasmid containing the first 557 bp of the HAdV-5 genome did not reveal major differences in E1A enhancer/promoter activity between cell types (Fig. S2). Late viral gene expression mirrored the kinetics of viral genome replication, as expected (Fig. 2C). We conclude that HAdV-5 replication and lysis are considerably more rapid in HBECs than in melanoma cells. Replication and lysis in lung cancer cells is, dependent on the cell line, rapid or intermediate. For other primary cells, replication kinetics were dependent on the cell type: epithelial keratinocytes showed rapid and mesenchymal fibroblasts, as reported before [27], [30], showed slow replication kinetics.

### Comparative analysis of HAdV-5-induced changes to cellular gene expression in cancer cells versus HBECs

We next investigated whether cellular gene expression is differently affected by Ad infection in cancer versus normal cells. Therefore, we performed a comparative analysis of HAdV-5 infection-induced changes in transcriptomes of HBECs (2 different donors), squamous cell lung cancer cells (SK-MES-1, SW900) and melanoma cells (SK-MEL-28, Mel624). The cells were cultured using standardized conditions and media (see Materials and Methods for details) and were infected with HAdV-5 at titers resulting in 80% transduction efficiency for each cell type or were mock-infected. Cells were harvested at the time point of onset of viral genome replication, because at that time key changes to the cellular transcriptome in preparation of viral DNA replication were expected. Furthermore, previous studies have shown less changes at earlier time points for other cells [27], [28], [30]. By choosing this time point for each cell type individually, according to the kinetics shown in Fig. 2, we could adjust for differences in viral replication kinetics between the cell types. Total RNA was purified and used for expression profiling. Microarray data are available on-line at the ArrayExpress database (accession number E-MEXP-3125). During bioinformatic analysis gene expression in uninfected samples was defined as steady state and compared to gene expression levels in infected samples. Thereby, we determined the virus-induced cellular transcriptome, i.e. infection-specific gene expression changes for each cell type individually without creating a bias through inter-cell type variations by different genetic backgrounds (see Materials and Methods for details).

We found the strongest changes in gene expression by HAdV-5 infection in HBECs, followed by lung cancer cells, whereas gene expression was much less affected by HAdV-5 infection in melanoma cells (see also correspondence analysis in Fig. S3). Both the numbers of cellular genes significantly regulated by HAdV-5 infection (Table 1) and the fold changes in gene expression (ArrayExpress, E-MEXP-3125) were highest for HBECs and were lowest for melanoma cells. These results correlate with the replication efficiency of HAdV-5 for these cells: HBECs showed both most efficient HAdV-5 replication and strongest virus-induced gene expression, whereas for melanoma cells both replication efficiency and virus-induced changes in gene expression were lowest.

**Table 1:** Number of genes regulated by HAdV-5 infection (p<0.05) in HBEC, SW900, SK-MES-1, SK-MEL-28 and Mel624.

cell line	number of regulated genes	upregulated	downregulated
HBEC	943	424	519
SW900	772	326	446
SK-MES-1	709	310	399
SK-MEL-28	314	112	202
Mel624	212	90	112

As expression profiling of Ad infection of normal respiratory epithelial cells has not been reported before, we first assessed the HAdV-5-induced cellular transcriptome in HBEC. Our results show the induction of genes involved in DNA replication and cell cycle, chromatin organization, and nucleotide metabolism, whereas genes involved in differentiation, regulation (mostly negative) of proliferation, and cell death regulation were repressed (Table 2 and Table S2).

**Table 2:** Gene annotations and genes significantly accumulating in the top 100 up-regulated genes and gene annotations significantly accumulating in the top 100 down-regulated genes in HBEC (see Table S2 for genes significantly accumulating in top 100 down-regulated genes in HBEC).

Upregulated GO-Terms	
GO-Term/gene	P-value/fold induction
**protein-DNA complex assembly**	**2,2E-3**
**chromatin assembly**	**3,3E-3**
**DNA packaging**	**6,1E-3**
**chromatin assembly or disassembly**	**7,2E-3**
H2BFS	9,29
HIST1H2	6,36
PRR6	5,05
HIST1H3	4,65
NAP1L5	4,44
HIST2H4	4,3
CHAF1B	3,93
**DNA replication**	**6,4E-3**
CCNE2	6,23
RRM2	5,2
CHAF1B	3,93
CDT1	3,7
CDC25A	3,45
MCM7	3,29
CDC45L	3,15
MCM10	2,88
**nucleosome assembly**	**7,2E-3**
**nucleosome organization**	**1,0E-2**
H2BFS	9,29
HIST1H2	6,36
HIST1H3	4,65
NAP1L5	4,44
HIST2H4	4,3
CHAF1B	3,93
**DNA metabolic process**	**1,8E-2**
CCNE2	6,23
RRM2	5,2
UNG	5,1
CHAF1B	3,93
CDT1	3,7
ALKBH2	3,6
CDC25A	3,45
MCM7	3,29
CDC45L	3,15
EXO1	3,12
MCM10	2,88

Downregulated GO-Terms	
GO-Term	P-value
**regulation of cell proliferation**	**3,7E-8**
**blood vessel morphogenesis**	**3,7E-6**
**blood vessel development**	**9,9E-6**
**vasculature development**	**1,0E-5**
**regulation of myeloid cell differentiation**	**4,4E-6**
**negative regulation of cell differentiation**	**1,8E-5**
**negative regulation of cell proliferation**	**5,3E-5**
**regulation of response to external stimulus**	**9,0E-5**
**response to mechanical stimulus**	**1,7E-4**
**regulation of apoptosis**	**1,8E-4**
**regulation of cell death**	**1,8E-4**

**Figure 3:**
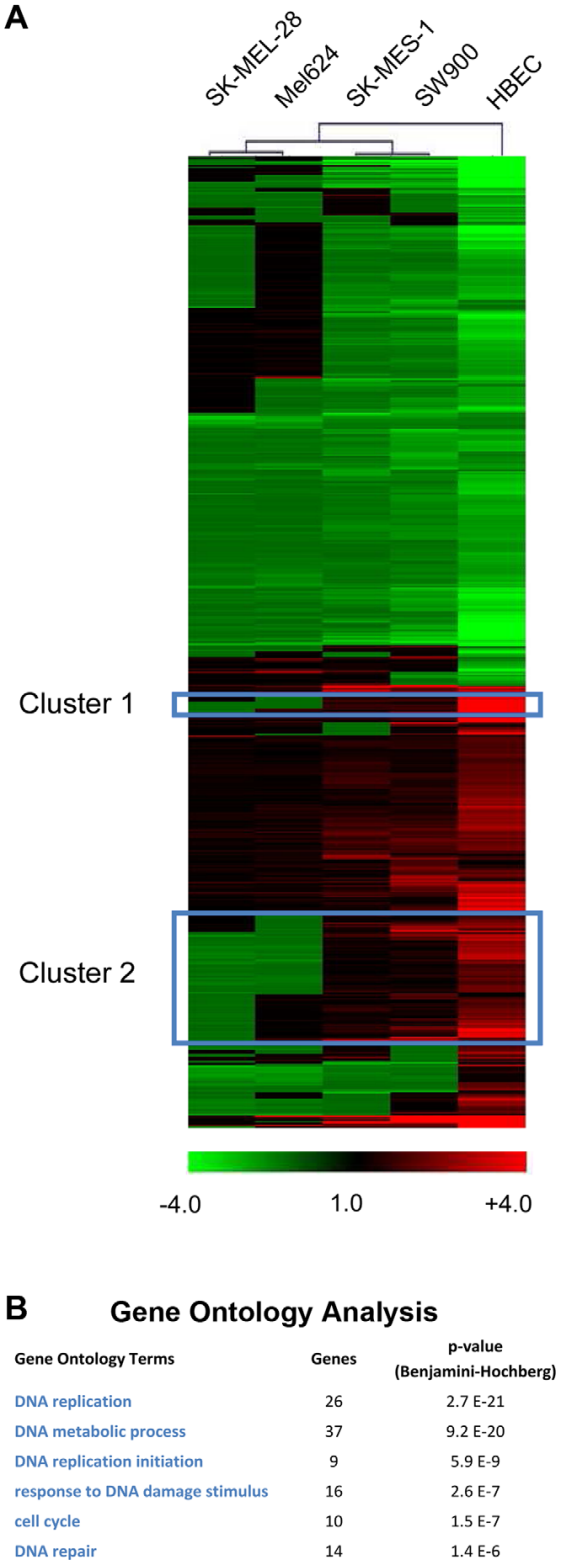
Hierarchical clustering of HAdV-5-induced gene expression in HBEC and tumor cells. (A) Hierarchical clustering of genes using Multi-Experiment Viewer (*MeV 4.5.1*) based on the approx. 1000 most significantly regulated genes (*see* *Materials and Methods* *for data filtration criteria*). Clusters of genes showing opposing regulation by HAdV-5 infection in HBEC versus melanoma cells are framed. Magnifications of these clusters are presented in Fig. S4. (B) Lists of genes within cluster 2 were subjected to gene ontology analysis using the Database for Annotation, Visualization and Integrated Discovery (*DAVID;* david.abcc.ncifcrf.gov). P-values of GO terms were corrected for multiple testing using the Benjamini-Hochberg algorithm.

Next, we compared gene expression signatures of HAdV-5 infection in HBECs and cancer cells by different bioinformatic analyses. Hierarchical clustering of differentially regulated genes of the five analyzed cell types highlighted two clusters of genes that show opposing regulation in melanoma cells versus HBECs, i.e. genes that are induced in HBECs, but repressed in one or both of the melanoma cell lines (framed in Fig. 3A, magnified in Fig. S4). Interestingly, the larger cluster shows a highly significant accumulation of genes involved in DNA replication, nucleotide metabolism, cell cycle regulation and DNA damage response (Fig. 3B), which were also found in the smaller cluster (here significance of accumulation was not reached because of the small number of genes). Selected genes are listed in Table 3. These cellular functions have been widely reported to be induced by Ad infection [17] and these clusters contain several of the genes most strongly induced in HBECs (see Table 2). Thus it is striking that HAdV-5 fails to induce or frequently even represses these genes/cellular functions in melanoma cells. The gene expression data obtained by microarray analysis was validated by quantitative PCR (Fig. S5). As a further bioinformatics approach to identify cellular pathways most differentially regulated by HAdV-5 infection of melanoma cells versus HBECs, we performed Ingenuity pathway analysis of the gene expression data. We identified the G1/S transition regulatory network with key pro-S phase genes (*E2F*, *CCNE*, *CDK2*, *Cdc25A*) induced in HBECs, but repressed or not regulated in melanoma cells (Fig. S6). We conclude that HAdV-5 infection of melanoma cells fails to induce a panel of S phase genes involved in cell cycle regulation, nucleotide metabolism and DNA replication and repair, which are induced in the native HAdV-5 cells, HBEC.

**Table 3:** Examples of genes with opposing regulation by HAdV-5 infection in HBECs versus melanoma cells.

gene symbol	regulation in fold change expression after infection			description
	HBEC	SW900/SK-MES-1	SK-MEL-28/Mel624	
E2F2	11.5	1.9/1.7	−1.5/(1.1)	transcription factor
MCM7	3.3	1.3/(1.1)	−1.5/(−1.2)	replication initiation
MCM10	3.3	1.2/1.2	−1.4/(−1.3)	replication initiation
CENPM	3.3	1.3/(−1.0)	−1.5/(−1.3)	centromere protein
CDC45L	3.2	1.3/(1.0)	−1.7/−1.6	DNA replication factor
RAD51AP	3.1	−1.4/(1.0)	−1.7/−1.7	DNA damage signaling
MCM2	3.0	1.4/(1.2)	−1.5/(−1.1)	replication initiation
PFS2	2.9	1.5/(1.2)	−1.4/(−1.2)	DNA replication factor
BLM	2.7	(1.2)/1.2	−1.6/−1.5	DNA helicase
TYMS	2.5	1.3/(1.0)	−1.5/(−1.2)	thymidylate synthase
MCM5	2.2	(1.3)/(1.1)	−1.6/(−1.2)	replication initiation
RFC4	2.1	(1.0)/(−1.0)	−1.5/−1.4	DNA replication factor
PRIM1	2.1	(−1.1)/(−1.1)	−1.9/−1.9	primase
CDK2	2.1	1.4/(1.1)	−1.3/−1.7	kinase
POLQ	2.1	(−1.1)/(1.1)	−1.5/−1.6	polymerase
BIRC3	1.5	(−2.7)/(−2.5)	(1.0)/(1.1)	apoptosis inhibitor
KIF2C	1.4	−1.3/−1.2	−2.0/−2.1	motor protein
TOP2A	1.4	−1.4/−1.5	−2.5/−2.2	topoisomerase
AURKB	1.4	(1.1)/(−1.2)	−2.1/−2.2	kinase
CENPF	1.2	(−1.1)/−1.3	−2.0/−2.1	centromere protein
CDKN1A	−2.9	(−1.2)/(−1.1)	(1.2)/3.1	kinase inhibitor
EGR1	−3.1	(1.0)/2.5	1.8/3.3	transcription factor
FOS	−4.7	(1.0)/1.3	1.7/2.0	transcription factor

Numbers in parentheses: p>0.05; note that maximal up-/down-regulation is 11.5/−16.1 for HBEC, 2.8/−3.3 for SK-MEL-28 and 4.2/−2.6 for Mel624.

### Analysis of S phase induction by HAdV-5 infection using the E2F-1 promoter as reporter

**Figure 4:**
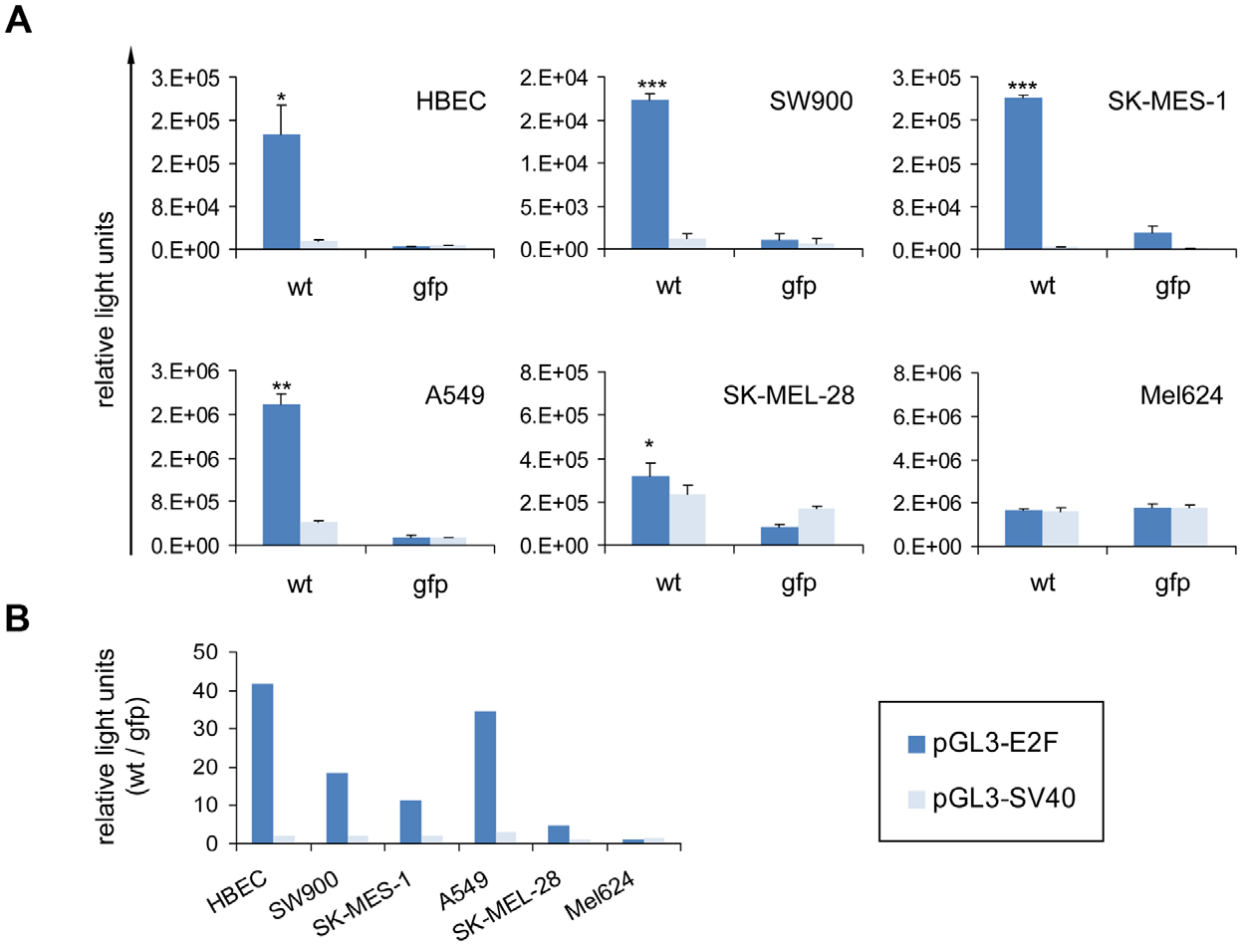
Analysis of S phase induction by HAdV-5 in HBEC and tumor cells using the E2F-1 promoter as reporter. (A) Luciferase reporter gene plasmids containing E2F-1 or SV40 promoter fragments were transfected into the indicated cell lines. After 24 hours, cells were infected with HAdV-5 (*wt*) or replication-deficient HAdV-5 CMV-gfp (*gfp*) at titers resulting in 80% infection. Luciferase activity was quantified twenty hours post infection. Columns represent mean values of triplicate transfections/infections and error bars reflect standard deviation. Asterisks indicate statistical significance for comparisons of HAdV-5 with HAdV-5 CMV-gfp (* p≤0.05, ** p≤0.01, *** p≤0.001). (B) Different representation of data shown in panel A: Fold change in promoter activity by HAdV-5 compared with HAdV-5 CMV-gfp.

As gene expression profiling indicated that HAdV-5 fails to induce genes critically involved in S phase processes in melanoma cells, we next performed an independent biological assay to investigate S phase induction by HAdV-5 infection. The E2F-1 promoter, which is strongly induced during S phase, was used as a reporter for Ad-induced S phase entry [31]. HBEC, SW900, SK-MES-1, A549, SK-MEL-28 and Mel624 cells were transfected with luciferase reporter plasmids containing either the E2F-1 promoter or the constitutive SV40 promoter as control. Transfected cells were subsequently superinfected with either HAdV-5 or with HAdV-5 CMV-gfp as E1-deleted, replication-deficient control virus. Quantification of reporter gene expression (Fig. 4) revealed that in lung cancer cells, infection with HAdV-5 induces the E2F-1 promoter, but not the SV40 promoter, much stronger than the replication-deficient virus control. In contrast, E2F-1 promoter induction by HAdV-5 infection was minimal or lacking in melanoma cells. These results are in accord with our gene expression profiling data and show that S phase induction by HAdV-5 is efficient in HBEC, but poor in melanoma cells.

## Discussion

Productive Ad infection is dependent on a cellular environment that supports the different stages of the viral replication cycle. Therefore, Ads have evolved strategies to manipulate the cellular environment in host cells. Immediate early and early viral proteins establish a complex network of interactions with host cell functions in order to counteract host defenses and induce cellular pathways necessary for replication of the viral genome. This includes the re-programming of cellular gene expression. Here, we describe for the first time the HAdV-5 infection-induced transcriptome for HBECs, representing their native host cells. We first determined the kinetics of HAdV-5 replication in HBECs, which is as follows: E1A expression starts before 4 hours post-infection (h.p.i.) and reaches a plateau at 8 h.p.i.; the first increase in viral genome and fiber mRNA copies occurs at 8 or 12 h.p.i., dependent on the donor, and infectious particle production reaches a plateau at 48 h.p.i.. This is followed by an efficient virus spread in the cell monolayer, as observed by cytotoxicity assay after low titer infection. Genome-wide expression profiling of HAdV-5-infected HBECs at the onset of viral genome replication, when major modifications to the host cell by early viral genes are expected, revealed a significant increase in expression for 424 and a decrease for 519 of 18,631 assessed genes. Thus gene repression was prominent at this time point, even considering that it is more difficult to detect than gene induction because of the half life of mRNA present before infection. Genes involved in the cell cycle, DNA replication, chromatin organization and nucleotide metabolism were accumulated in the upregulated gene population. This included *E2F-2*, showing the strongest increase in mRNA expression (11.5-fold), *CCNE1* and *CCNE2* (cyclin E1 and E2; 7.9- and 6.2-fold), *RRM2* (ribonucleotide reductase M2; 5.2-fold), *CDC25A* (3.5-fold), *MCM2*, *7* and *10* (3-, 3.3- and 3.3-fold), *EXO1* (exonuclease 1; 3.1-fold), *RFC3* and *4* (replication factor C3 and 4; 2.8- and 2.1-fold), *PCNA* (proliferating cell nuclear antigen; 2.2-fold) and several histone, chromatin assembly factor and centromere genes. These results are in accord with previous studies which have established, though in other cell types, that S phase induction in Ad-infected cells is required for viral replication to proceed. The number of genes and induction rates that we report might be even underestimated, as we did not synchronize HBECs before infection in order to allow for a valid comparison to cancer cells. Genes with activity in differentiation, negative regulation of proliferation (including *CDKN1A*; −2.9 and *CDKN2B*; −2.73), and cell death regulation were accumulated in the repressed gene population (strongest repression was for parathyroid hormone-like hormone, 16.1-fold). Of note, we did not identify the accumulation of genes involved in immunity in the upregulated gene pool.

HAdV-5 infection has been rarely investigated in normal respiratory epithelial cells before. Several studies of oncolytic Ads have compared primarily respiratory epithelial cells with tumor cells in cytotoxicity and infectious particle production assays to assess the selectivity and efficiency of virus mutants [32]–[39]. These studies have shown that the production of infectious HAdV-5 particles in primary respiratory epithelial cells is efficient and peaks around 48 h p.i. [37], [39], which is in accord with our results.

Previous studies on expression profiling of Ad infection have been performed with HeLa cells and human fibroblasts. For HeLa cells, HAdV-2 infection at MOI 100 was reported to regulate 76, 60, or 382 genes more than 1.5-fold at 6 h, 10 h or 20 h.p.i., respectively (12,000 were analyzed at 6 h, 7,500 genes at 10 h and 20 h) [26], [28]. Another study found 75 of 4,600 genes regulated more than twofold by HAdV-5 at 24 h.p.i. of HeLa cells [40]. Whether the lower number of regulated genes in comparison to HBECs in our study is due to the transformation of HeLa cells is difficult to judge because of differences in methodology. In this regard, our study showed a lower number of regulated genes for both lung cancer and melanoma cell lines in direct comparison with HBECs (see below). S phase genes were reported to be induced by Ad infection in HeLa cells, including *CDC25A* (1.8 at 10 h), *UNG* (1.6) and histone genes [28], which we found also in HBECs. Overlaps are also found in downregulated genes: *MYC* (−1.9 versus −2.4 in HBEC); *THBS1* (thrombospondin-1; −2.6 versus −9.4 in HBEC); *CAV2* (caveolin-2; −1.4 versus −4.0 in HBEC). In fibroblasts, transcriptomes were reported over a period of two days after infection of synchronized cultures with HAdV-2 at MOI 100 [27], [29], or after infection with HAdV-5 at MOI 30 [30]. Zhao and co-workers investigated an array of 29,300 cDNA clones and observed that 190 genes were upregulated and 265 genes were downregulated at 24 h.p.i., when viral genome replication initiated [27]. This is lower in number but similar in ratio to our findings in HBECs. Genes involved in cell cycle, proliferation and DNA metabolism were upregulated including several genes that we also identified in HBECs: *CDC25A* (3.2); *CCNE1* (2.3), *CCNE2* (4.0); *MCM3*, *4*, *5*, and *6* (2.7, 4.7, 7.0, and 3.1); *PCNA* (7.3); *EXO1* (2.9); *RFC3* and *4* (2.4 and 2.5) and several histones. Downregulated genes included differentiation factors, cytoskeleton proteins and vesicle transport proteins with *VEGF*, *VEGFC*, *syntaxin 6*, *THBS1*, collagen and claudin genes also found in our study for HBECs. Miller and co-workers report 2,104 of 20,590 genes regulated more than twofold during the 2 day interval investigated, two thirds of which were upregulated [30]. This study identified at the onset of viral replication in the upregulated gene pool a significant accumulation of gene ontology terms DNA replication, cell cycle, mitosis, M phase, DNA repair, chromatin assembly, nucleosome assembly, RNA splicing, intracellular transport, nucleocytoplasmic transport and ribosome biogenesis, including several E2F-responsive genes that we also found in HBECs. Overall our study identified a transcriptome for HAdV-5 in HBECs with primarily S phase genes induced, which correlates with previous observations in HeLa cells and fibroblasts.

Our study highlights that Ad replication can be attenuated in tumor cells versus their native host cells. Specifically, we observed a delay in E1A expression, onset of DNA replication and late gene expression for HAdV-5 in tumor cells compared with HBEC. This was most pronounced for Mel624 cells and least for SW900 cells. Indeed the viral replication kinetics in melanoma cell lines resembled those of fibroblasts, for which a slow Ad replication cycle has been shown previously [29], [30]. Spread-dependent lytic activity of SW900 was similar to HBECs, but attenuated in SK-MES-1 and even more so for the melanoma cell lines, in spite of their higher susceptibility to transduction by HAdV-5 vectors. As primary keratinocytes resembled HBECs with respect to HAdV-5 replication kinetics and spread-dependent cytotoxicity, our data indicate that HAdV-5 replication is delayed in non-epithelial versus epithelial cells. It is notable that HAdV-5 showed the most rapid replication kinetics in HBEC followed by normal keratinocytes even when compared with epithelial lung cancer cell lines. Hence, cellular transformation does not necessarily increase the permissivity for Ad infection. From our data it can be concluded that the timing of expression of the immediate early gene E1A is a major determinant of cell type-dependent differences in HAdV-5 replication kinetics. The reasons for the cell type-dependent differences in E1A expression remain to be determined. We observed only minor differences in E1A enhancer/promoter activity after transient transfection. Moreover, we previously observed that overexpression of E1A from the CMV enhancer/promoter does not increase lytic activity of HAdV-5 mutants in SK-MEL-28 cells [41]. These results argue that cell type-dependent differences in viral uptake, intracellular virus trafficking, nuclear translocation of the virus genome and/or mRNA stability might cause differences in E1A expression kinetics and subsequent lytic activity of HAdV-5.

The considerable differences in the kinetics of early viral gene expression make clear that it is important to look at the same stage of the viral replication cycle (rather than the same time point post infection) when comparing host cell responses in different cell types. Therefore, we performed our comparative analysis of the HAdV-5-induced transcriptome at the onset of viral genome replication. The number and fold induction rates of HAdV-5-induced cellular genes was lower for tumor cell lines in the order SW900, SK-MES-1, SK-MEL-28 and Mel624, which matched the kinetics and efficiency of viral replication in these cells. In all cell types, less genes were induced (36% to 45% of all regulated genes) than repressed. HAdV-5-induced transcriptomes of the squamous cell carcinoma lines were more similar to HBEC than those of the melanoma cell lines. A clear qualitative difference in the HAdV-5 induced transcriptome, however, was observed between melanoma cells and HBEC, represented by two clusters of genes repressed in melanoma cells, but induced in HBEC. Such differences cannot be explained by the delay in E1A expression in melanoma cells and must result from other host cell-dependent factors. Especially genes involved in cell cycle, DNA replication and DNA metabolism were accumulated in the larger of the two clusters with high significance, indicating that HAdV-5 fails to induce S phase genes in these melanoma cells. Also the smaller cluster contained major S phase genes. However, significance for accumulation of certain gene annotations was not reached for this cluster due to its small size. Key player genes in cell cycle control and DNA replication are included in these clusters: *E2F2* (−1.5-fold in SK-MEL-28 versus 11.5-fold in HBEC), *CCNE* (−1.8/3.2), *CDC45L* (−1.7/3.3), *MCM2* (−1.5/3.0), *BLM* (−1.6/2.7); note that maximal repression in SK-MEL-28 was 3.3-fold. These expression profiling results are supported by a drastically or even completely reduced induction of the E2F-responsive E2F-1 promoter, a reporter for S phase induction, in melanoma cells compared with HBEC and lung cancer cells. As productive Ad replication is dependent on the host cell entering S phase, these differences are striking and likely to contribute to the delay in virus replication and the reduced cytotoxicity of HAdV-5 in melanoma cells. It is important to consider that in the chosen experimental conditions, both HBECs and tumor cells were cultured in low serum conditions and were proliferating. Thus melanoma cells should enter S phase eventually. Still, HAdV-5 infection could accelerate S phase entry in HBEC, but less so in melanoma cells. Furthermore, virus-induced S phase entry might be of a different quality than S phase entry within a normal cell cycle. Our efforts to identify, in a larger panel of melanoma cells, individual virus-induced genes as predictors of HAdV-5 oncolytic efficacy were not successful, indicating that it is important to look at modifications of gene networks rather than individual genes.

Based on this study it will now be of interest to elucidate the molecular basis for both the delayed replication kinetics and the different gene expression signature of HAdV-5 infection in melanoma cells compared with HBECs. Specifically, host factors should be identified that differentially determine the following parameters: kinetics of virus uptake, intracellular virus trafficking, nuclear translocation of the virus genome, viral gene expression, and/or viral manipulation of cellular gene expression. Such studies should be extended to other tumor types because we hypothesize that especially tumors of non-epithelial origin, like sarcomas or lymphomas, show attenuated and/or delayed Ad replication. HAdV-5-induced transcriptomes of these cells might reveal differences to HBECs which could be either similar or distinct to our results for melanoma cells. Ultimately, these efforts will provide opportunities for the development of optimized oncolytic Ad therapies. Scenarios towards this end include the genetic modification of oncolytic Ad genomes in order to complement infection-supportive host cell activities that are lacking in target tumor cells. Alternatively, such activities could be induced by combination therapy, for example by addition of S phase-inducing chemotherapies. Finally, directed evolution of Ads in tumor cells might provide Ad mutants that establish an improved replication efficacy and lytic activity based on accelerated E1A expression and/or improved induction of S phase genes.

## Materials and Methods

### Cell culture

Human cell lines A549, SK-MEL-28, SW900 (all ATCC, Manassas, VA), Mel624 (kindly provided by J. Schlom, Bethesda, MD) and SK-MES-1 (cell repository German Cancer Research Centre, Heidelberg) were all maintained in DMEM. 293 cells (QBiogene, Heidelberg, Germany) were cultivated in RPMI1640. HFF cells (primary human foreskin fibroblast; kindly provided by M. Marschall, Erlangen, Germany) were cultivated in MEM (Invitrogen, Karlsruhe, Germany). Media were supplemented with 10% heat-inactivated fetal bovine serum (FBS, PAA, Cölbe, Germany), 100 U/ml penicillin and 100 µg/ml streptomycin (both Invitrogen). Primary HBEC (Lot 5092901.17 derived from a 55 year old Caucasian male; Lot 7110910.11 originated from a 67 year old Caucasian male, both PromoCell, Heidelberg, Germany) as well as PHK cells (primary human keratinocytes from foreskin, kindly provided by N.S. Banerjee, University of Alabama at Birmingham, Birmingham, AL) were cultivated in complete Airway Epithelial Cell Growth Medium or Keratinocyte Growth Medium 2 (both PromoCell), respectively. Cells were grown at 37°C in a humidified atmosphere of 5% CO_2_. Media were pre-warmed to 37°C in a water bath before use.

### Sub-cultivation of permanent cell lines for viral gene expression, DNA replication, and microarray experiments

SK-MEL-28, Mel624, SW900 and SK-MES-1 were slowly adapted to low serum conditions. Therefore, FBS content was gradually reduced by mixing DMEM/10% FBS with rising volumes of Melanocyte Growth Medium (SK-MEL-28, Mel624) or Airway Epithelial Cell Growth Medium (SW900 and SK-MES-1). Finally, cells were sub-cultivated in a mixture of one volume DMEM/10% FBS and three volumes of Melanocyte Growth Medium or Airway Epithelial Cell Growth Medium, respectively (referred to as “microarray growth medium” herein). Frozen aliquots of 1×10^6^/ml adapted cells were stored in liquid nitrogen until further use. Prior to experiments, stocks were thawn in a waterbath at 37°C for one minute, transferred to a 15 ml falcon tube, and washed once in their respective microarray growth medium. Afterwards, approx. 16,000 cells/cm^2^ were seeded in pre-warmed and CO_2_ equilibrated microarray growth medium. The next day, medium was exchanged and cells incubated for another day.

### Recombinant adenoviruses

HAdV-5 is wild-type human adenovirus serotype 5. HAdV-5 CMV-gfp is an E1/E3-deleted gene transfer vector derived from HAdV-5 that contains a CMV promoter-driven GFP gene. Viruses were amplified by serial passages in A549 cells (HAdV-5) or 293 cells (HAdV-5 CMV-gfp) and were purified by two rounds of CsCl equilibrium density gradient ultracentrifugation. Verification of viral genomes was performed by PCR. Physical particle concentration (viral particle (vp)/ml) was determined by OD_260 nm_ reading; infectious viral particle titers were determined by TCID_50_ assay on 293 cells.

### Virus-mediated spread and cytotoxicity

To determine virus mediated cytotoxicity, 3×10^4^ cells were seeded in 48 well plates. The following day, cells were infected in 200 µl DMEM/2% FBS containing Ads with concentrations from 0.0001–10 TCID_50_/cell in tenfold serial dilutions or were mock-infected. The next day, 500 µl growth medium was added and cells incubated until cytopathic effects could be observed in wells containing a low viral inoculum. Cell lysis was documented by aspirating the cell culture supernatants and staining of live cells by addition of 100 µl of 2% crystal violet in 70% ethanol for 30 minutes at room temperature. Afterwards, plates were rinsed twice in water to remove excessive dye, air-dried, and scanned to obtain digital images.

### Detection of CAR expression by FACS analysis

1×10^6^ cells were incubated on ice for 2 h with 14 µg/ml mouse anti-CAR antibody RmcB (supernatant of hybridoma) or IgG_1_ isotype control, clone MOPC-21 (BioLegend, San Diego, CA) in FACS buffer (PBS, 10% FCS and 0.01% NaN_3_). Cells were washed twice with 1 ml FACS buffer and the incubated with 0.5 µg/ml anti-mouse-PE secondary antibody (BD Pharmigen, San Diego, CA) for 1 h on ice. For final read-out, cells were washed twice with 1 ml FACS buffer and resuspended in 600 µl FACS buffer. Cells were analyzed by flow cytometry (FACSort, BD Biosciences, Heidelberg, Germany) and data was evaluated using FCS Express Version 3 software (De Novo Software, Los Angeles, CA).

### Determination of transduction rates in living cells

To determine transduction efficiency of HAdV-5 for each individual cell type, 3×10^4^ cells were seeded into 24 well plates and transduced after two days with various TCID_50_ titers of HAdV-5 CMV-gfp diluted in 250 µl microarray growth medium or DMEM/2% FBS (A549, PHK and HFF). After one hour at 37°C, medium was aspirated and replaced by 500 µl fresh microarray growth medium or growth medium (A549, PHK and HFF). Cells were harvested 48 hours post infection by trypsinization and transgene levels in living cells were measured by flow cytometry. To this end cells were washed twice in FACS washing buffer DPBS/1% (v/v) FBS/0.01% (v/v) sodium azide and collected by centrifugation at 225 g for 3 minutes. For staining of dead cells, pellets were resuspended in 100 µl DPBS containing a final concentration of 50 µg/ml propidium iodide and 100 µg/ml RNAse A. After incubation at room temperature for 10 minutes, cells were further diluted in 200 µl FACS buffer and analyzed immediately. All samples were analyzed on a FACScan machine (BD Biosciences). Appropriate compensation was set up for each experiment individually and data was recorded for at least 10,000 events. Data was analyzed using FCS Express Version 3 software.

### DNA/RNA quantification by real-time PCR or microarray analysis

For quantification of viral genome copy numbers or RNA expression, 3×10^4^ cells were seeded in 24-well plates. For microarray experiments samples were upscaled accordingly to 6 well plates. After two days, cells were infected in 250 (1000 for 6 well) µl microarray growth medium for one hour. Thereafter inoculants were removed and samples were harvested at indicated time points. Total genomic DNA was purified from cell lysates with the QIAamp BloodMiniKit (Qiagen, Hilden, Germany); RNA was purified with the RNeasy kit (Qiagen) following the manufacturer's instructions. For microarray analysis, QIAshredder® columns (Qiagen) were utilized to ensure higher lysate homogeneity. For qPCR samples only, a digest with a RNAse free DNAse Kits (Qiagen) was performed on the column or after column purification according to the manual. Oligonucleotides used for quantification of viral genomes, viral E1A, fiber and hexon mRNA, cellular DNA and cellular RNA were described in. Oligonucleotides for quantification of cellular genes were obtained from Qiagen, Hilden and reconstituted in 1.1 ml TE buffer, pH 8.0. Following primers were used: ACTB QT01680476, BLM QT00027671, CCNE1 QT00041986, CD83 QT00069923, CDC25A QT00001078, CDT1 QT00020601, CHAF1B QT00012845, E2F2 QT00045654, E2F5 QT00062965, EGR1 QT00999964, FOS QT00007070, GAPDH QT01192646, H2BFS QT00227199, HERC5 QT00007280, HES4 QT00208726, IRS2 QT00064036, MCM2 QT00070812, MGC13057 QT00221347, PKMYT1 QT00013580, RFC3 QT00019243, TIPIN QT00054334, UNG1 QT00037912. Adenoviral genome copies, viral mRNA as well as cellular mRNA expression levels were quantified by qPCR based on the detection of Sybr Green on a 7300 Real Time PCR System (Applied Biosystems, Darmstadt, Germany) using MicroAmp® 96 Well Reaction Plates (Applied Biosystems). Each 25 µl sample contained 1× Power SYBR® Green PCR Master Mix, 50 U/µl reverse transcriptase (for RNA templates only), 20 U/µl RNAse inhibitor (for RNA templates only), 10 µM of each primer or 1× Quantitect™ primer mix and 20 ng template. For mRNA quantification reverse transcription was carried out in plates at 48°C for 30 minutes directly before qPCR was performed with an initial denaturation step of 10 min at 95°C, followed by 40 cycles of 15 s denaturation at 95°C and 1 minute of annealing and elongation at 60°C. At the end of each cycle, the fluorescence emitted by the SYBR Green was measured. After completion of the cycling process, samples were subjected to an optional melting curve analysis from 60°C to 95°C at 0.1°C/s with continuous fluorescence monitoring to distinguish primer dimers and unspecific amplicons from specific target gene products. Each run further included negative controls as well as appropriate standard curves whenever available. Standard curves for quantification of the copy numbers of viral genomes or viral mRNA was diluted from pTG3602 plasmid (10^8^, 10^6^, 10^4^ and 10^2^ copies/µl). Data was normalized with cellular genomic DNA (determination of viral genome copy numbers) or cellular RNA (determination of viral mRNA copy numbers) for each sample individually. Cellular RNA was quantified using GAPDH oligonucleotides and 200, 20, 2 and 0.2 ng/µl of HeLa total RNA (Stratagene, Amsterdam, The Netherlands). Cellular DNA was quantified using β-actin oligonucleotides and 200, 20, 2 and 0.2 ng/µl human DNA isolated from A549 cells as standard. Data was analyzed with the 7300 System SDS Software V1.4 (Applied Biosystems) and presented as viral gene copy numbers and viral genome copy numbers normalized with cellular genomic DNA or cellular RNA for each sample individually. For cellular mRNAs, qgene was used to obtain mean normalized expression values [42].

### Burst assay

Experiments were carried out in triplicates using 3×10^4^ cells in 24 well plates but were otherwise identical as described for DNA/RNA quantification. In addition, virus inoculum was removed one hour post infection. Cells and supernatants were harvested at given time points and viruses were released from cells by three freeze thaw cycles. Cellular debris was removed by centrifugation and infectious virus particles in supernatants were quantified by TCID_50_ assays on 293 cells.

### Probe Labeling and Illumina Sentrix BeadChip array Hybridization

Total RNA from uninfected and infected cells was isolated. Labeling and hybridizations were performed at the Genomics & Proteomics Core Facility at the DKFZ. Briefly, sample quality was assessed by gel analysis using the total RNA Nano chip assay on an Agilent 2100 Bioanalyzer (Agilent Technologies, Berlin). Only samples with a RNA index value greater than 8.5 were selected for expression profiling. Biotin-labeled cRNA samples for hybridization on Illumina Human Sentrix-8 BeadChip arrays (Illumina, San Diego, CA) were prepared according to Illumina's recommended sample labeling procedure based on the modified Eberwine protocol [43]. In brief, 250–500 ng total RNA was used for cDNA synthesis, followed by an amplification/labeling step (*in vitro* transcription) to synthesize biotin-labeled cRNA according to the MessageAmp II aRNA Amplification kit (Ambion, Austin, TX). Biotin-16-UTP was purchased from Roche Applied Science, Penzberg, Germany. The cRNA was column purified according to TotalPrep RNA Amplification Kit, and eluted in 60–80 µl of water. Quality of cRNA was controlled using the RNA Nano Chip Assay on an Agilent 2100 Bioanalyzer and spectrophotometrically quantified. Hybridization was performed at 58°C, in GEX-HCB buffer (Illumina) at a concentration of 100 ng cRNA/µl, unsealed in a wet chamber for 20 hours. Spike-in controls for low, medium and highly abundant RNAs were added, as well as mismatch control and biotinylation control oligonucleotides. Microarrays were washed once in High Temp Wash buffer (Illumina) at 55°C and then twice in E1BC buffer (Illumina) at room temperature for 5 minutes (in between washed with ethanol at room temperature). After blocking for 5 min in 4 ml of 1% (w/v) Blocker Casein in phosphate buffered saline Hammarsten grade (Pierce Biotechnology, Rockford, IL), array signals were developed by a 10 minute incubation time in 2 ml of 1 µg/ml Cy3-streptavidin (Amersham Biosciences, Buckinghamshire, UK) solution and 1% blocking solution. After a final wash in E1BC, the arrays were dried and scanned.

### Scanning and data analysis

Microarray scanning was done using a Beadstation array scanner, adjusted to a scaling factor of 1 and PMT settings at 430. Data was extracted for all beads individually, and outliers were removed if >2.5 MAD (median absolute deviation). All remaining data points were used for the calculation of the mean average signal for a given probe, and standard deviation for each probe was calculated. Data were loaded into the Multi-Conditional Hybridization Intensity Processing System (M-CHiPS) [44] as two-channel data with the infected samples as test and the corresponding uninfected samples as reference channels, thereby eliminating variations between different cell lines. Data was subsequently normalized applying the locally weighted scatterplot smoothing algorithm, LOWESS [45]. Genes with ≥1.5-fold regulation and signal intensity >100 in at least one condition were considered for downstream analysis. To filter for reproducible expression changes between replicates, outliers were excluded using a very restrictive ‘min-max separation’ filter [46]. P-values for each condition versus the control condition were calculated using the empirical Bayes method in the LIMMA package (Bioconductor) [47]. Data were visualised in a correspondence analysis (CA) plot [48] allowing for simultaneous presentation of experimental conditions and genes (Fig. S3). Differences between conditions as well as genes associated with certain conditions are represented by their relative position within the CA plot. Differentially expressed genes were subjected to further analysis by hierarchical clustering using the MultiExperiment Viewer 4.3. Ingenuity Pathway Analysis (IPA; Ingenuity Systems, USA) and DAVID [49], [50] were used to find pathways or gene ontology terms exhibiting a significant number of differentially regulated genes. All gene expression data is MIAME compliant and the raw data has been deposited in the MIAME compliant ArrayExpress data base (http://www.ebi.ac.uk/arrayexpress) with accession number E-MEXP-3125.

### Reporter gene assay for S-phase entry

Twenty four hours prior transfection, 5–7.5×10^4^ cells (depending on the cell line) were seeded out in 0.5 ml of their respective growth medium in 24 well plates. Cells reached approximately 60–80% confluence on the next day and were transiently transfected with a pGL3 luciferase reporter plasmid containing either the SV40 promoter (Promega, Madison, WI) or the −221/+60 bp fragment of the human E2F-1 promoter in triplicates using Lipofectamine™ and PLUS™ reagent (both Invitrogen) as suggested by the manufacturer. Briefly, for every sample 0.5 µg DNA was mixed with 100 µl OptiMEM® containing 0.5 µl PLUS™ reagent and incubated at room temperature for 15 minutes. Then, 2.5 µl Lipofectamine™ was added and samples mixed by vortexing. Following another 30 minutes incubation time at room temperature, the transfection mix was added directly to the cells over night for 24 hours. Then, cells were infected with HAdV-5 or HAdV-5 CMV-gfp diluted in the respective growth medium containing 2% FBS or no fetal calf serum (HBEC). Inoculums were removed after one hour at 37°C and cells grown in their normal growth media for twenty hours before luciferase expression was quantified using the Luciferase Assay System (Promega). Therefore, cells were washed once with DPBS and lysed in 200 µl Reporter Lysis buffer. After incubation for at least 30 minutes at −80°C, lysates were thawed at room temperate. For each sample, 50 µl cell lysate was mixed with 50 µl Luciferase Assay substrate and immediately measured in a FluoroskanAsentFL (Thermo-Scientific, Braunschweig, Germany). Cells devoid for luciferase expression were used as negative controls to determine nonspecific background.

### Statistical analysis

Differences between indicated groups were analyzed using the Student's *t* test. For microarray data, P-values for each condition versus the control condition were calculated using the empirical Bayes method in the LIMMA package (Bioconductor) [47]. P-values for genes of microarray experiments and gene ontology terms were corrected for multiple testing using the Benjamini-Hochberg algorithm [51]. P-values of <0.05 were considered statistically significant.

## Supporting Information

Figure S1 **Expression of HAdV-5 receptor CAR on SK-MEL-28, Mel624, A549 and HFF cells.** Detection of CAR expression by flow cytometry after staining of cells with anti-CAR antibody RmcB (black line) or isotype control (grey).
(TIF)

Figure S2 **Reporter gene assay of E1A promoter activity in HBEC, SW900, SK-MES-1, SK-MEL-28 and Mel624.** Cells were transfected with luciferase reporter gene plasmids. Plasmids contained either no promoter (*pGL3-basic*, negative control), the E1A promoter (*pGL3-E1A*), the strong CMV promoter (*pGL3-CMV*), or the weak human thymidine kinase promoter (*pGL3-hTK*). Luciferase activity was quantified 48 hours post transfection and expressed as relative light units. Columns represent mean values of triplicate transfections and error bars reflect standard deviation; p-values were calculated for comparisons of the E1A and CMV promoter and for comparisons of the E1A and hTK promoter using the Student's t-test (* p≤0.05, ** p≤0.01).
(TIF)

Figure S3 **Correspondence analysis of HAdV-5 infection-induced transcriptomes of HBEC, SW900, SK-MES-1, SK-MEL-28 and Mel624.** After defining uninfected samples as steady state and filtering for significantly regulated genes (as described in Materials & Methods), a correspondence analysis plot was calculated in M-CHiPS. It simultaneously displays hybridizations (conditions, colored symbols) and genes (grey dots). The relative position of conditions indicates their similarities or differences. Conditions clustering closely together exhibit similar gene expression patterns while conditions located opposite of the centroid (0/0) exhibit substantial differences in gene expression. Genes strongly associated with a condition are located in the same direction from the centroid. Uninfected controls for all cell types are clustering because of the normalization procedure. The plot shows most substantial changes in cellular gene expression by HAdV-5 infection for HBEC (highest distance to uninfected control), followed by SW900 and SK-MES-1, but modest changes, only, for SK-MEL-28 and Mel624.
(TIF)

Figure S4 **Hierarchical clustering of HAdV-5-induced gene expression in HBEC, SW900, SK-MES-1, SK-MEL-28 and Mel624 reveals clusters with opposing regulation in HBEC versus melanoma cells.** Magnified clusters 1 and 2 from Fig. 3.
(TIF)

Figure S5 **Validation of microarray data by qPCR.** For a set of 7 selected genes, qPCR quantification of mRNA was performed. Concentrations of mRNAs were normalized to concentrations of ACTB based on qgene and values for uninfected HBEC were set to 1 to allow for comparison of qPCR and microarray data. Scatter blots show correlation of log_2_ transformed values from qPCR and microarray for uninfected cells (upper panel) and infected cells (lower panel). The coefficient of determination (R^2^) was calculated for each cell line based on linear trend curves with R^2^ = 1 equaling 100%. Analysis of this low number of genes already demonstrated a high correlation of qPCR and microarray data for HBEC, SW900, SK-MES-1 and SK-MEL-28. With this set of genes lower R^2^ values were obtained for Mel624.
(TIF)

Figure S6 **Pathway analysis of HAdV-5 infection-induced genes in HBEC versus SK-MEL-28.** Expression values were uploaded to the Ingenuity Pathway Analysis (IPA) software and mapped onto the canonical G1/S transition regulatory network of the cell cycle for HBEC and SK-MEL-28. Green and red nodes represent down- and up-regulated genes, respectively. Color intensity correlates with strength of fold change.
(TIF)

Table S1 **Infectious particle titers used to achieve 80% tranduction efficiency for individual cell types as determined by transduction with HAdV-5 CMV-gfp and quantification of GFP positive cells in the living cell fraction by fluorescence cytometry.**
(DOC)

Table S2 **Gene annotations and genes significantly accumulating in the top 100 down-regulated genes in HBEC.**
(DOC)
