# Supplementary figures and images for: Replication and Virus-Induced Transcriptome of HAdV-5 in Normal Host Cells versus Cancer Cells - Differences of Relevance for Adenoviral Oncolysis
Source: PLoS One. 2011 Nov 30;6(11):e27934. doi: 10.1371/journal.pone.0027934 (PMC3227638; doi:10.1371/journal.pone.0027934)

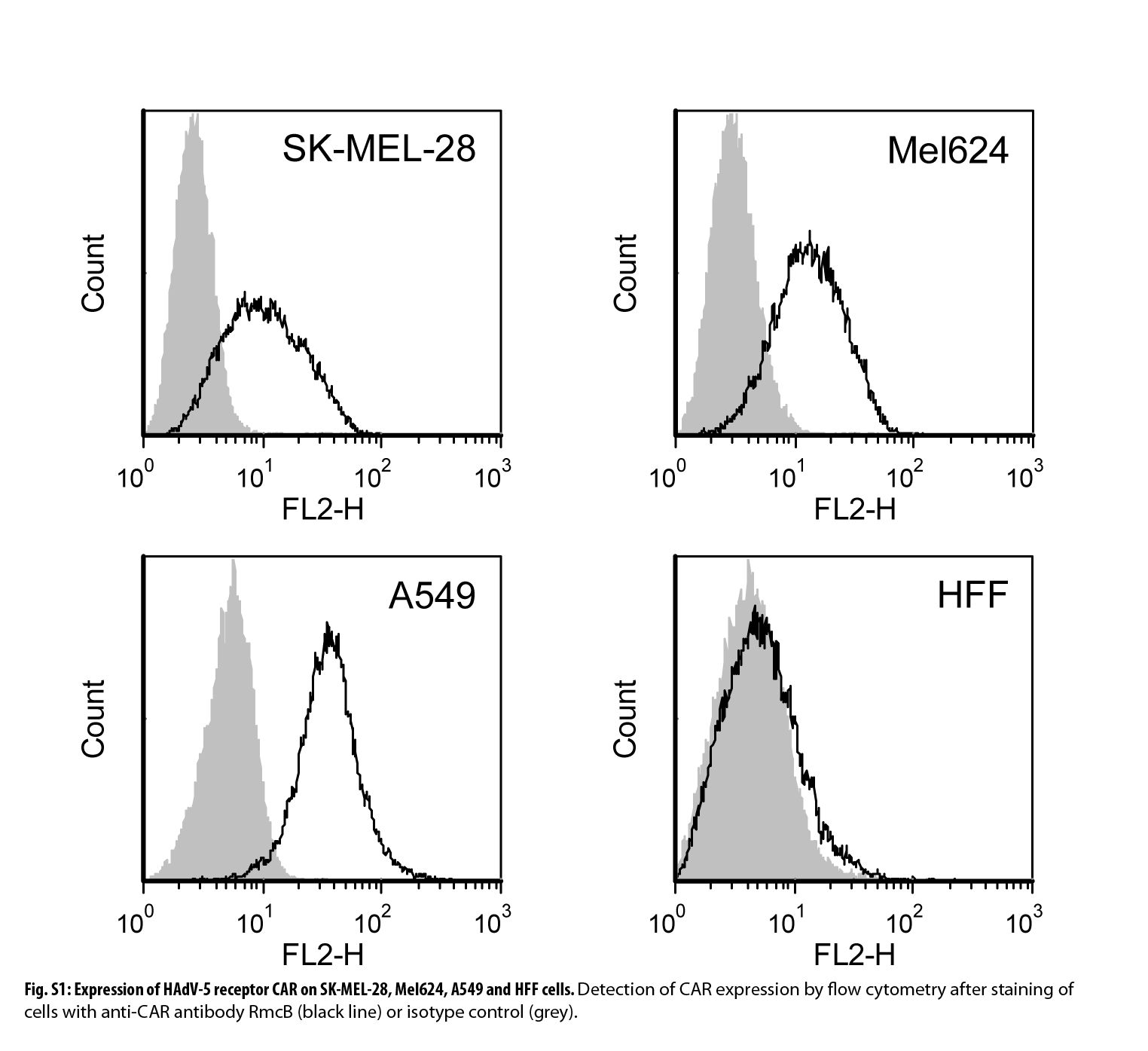

Supplement: Figure S1 — Expression of HAdV-5 receptor CAR on SK-MEL-28, Mel624, A549 and HFF cells. Detection of CAR expression by flow cytometry after staining of cells with anti-CAR antibody RmcB (black line) or isotype control (grey). (TIF) [file pone.0027934.s001.tif]

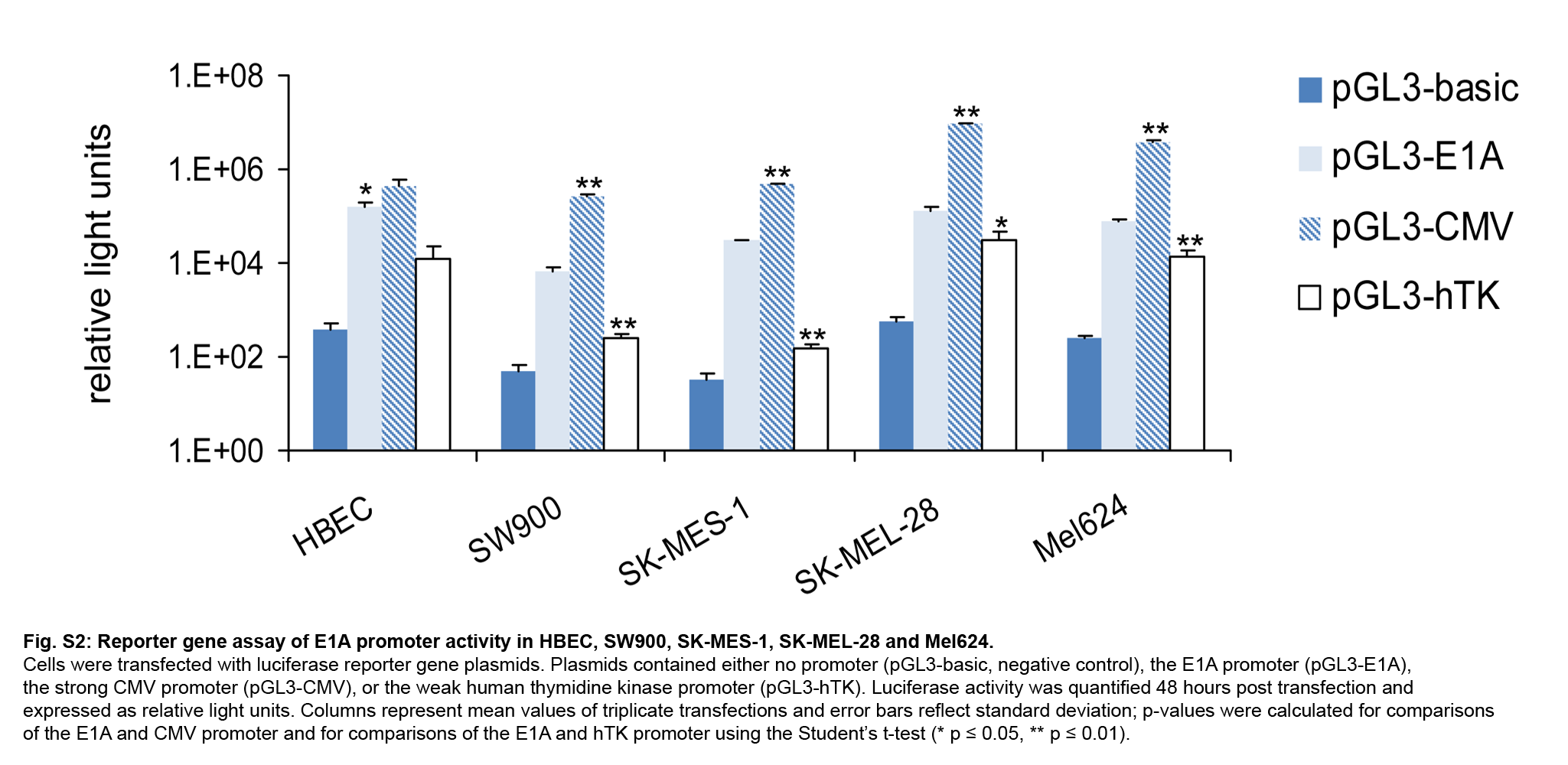

Supplement: Figure S2 — Reporter gene assay of E1A promoter activity in HBEC, SW900, SK-MES-1, SK-MEL-28 and Mel624. Cells were transfected with luciferase reporter gene plasmids. Plasmids contained either no promoter (pGL3-basic, negative control), the E1A promoter (pGL3-E1A), the strong CMV promoter (pGL3-CMV), or the weak human thymidine kinase promoter (pGL3-hTK). Luciferase activity was quantified 48 hours post transfection and expressed as relative light units. Columns represent mean values of triplicate transfections and error bars reflect standard deviation; p-values were calculated for comparisons of the E1A and CMV promoter and for comparisons of the E1A and hTK promoter using the Student's t-test (* p≤0.05, ** p≤0.01). (TIF) [file pone.0027934.s002.tif]

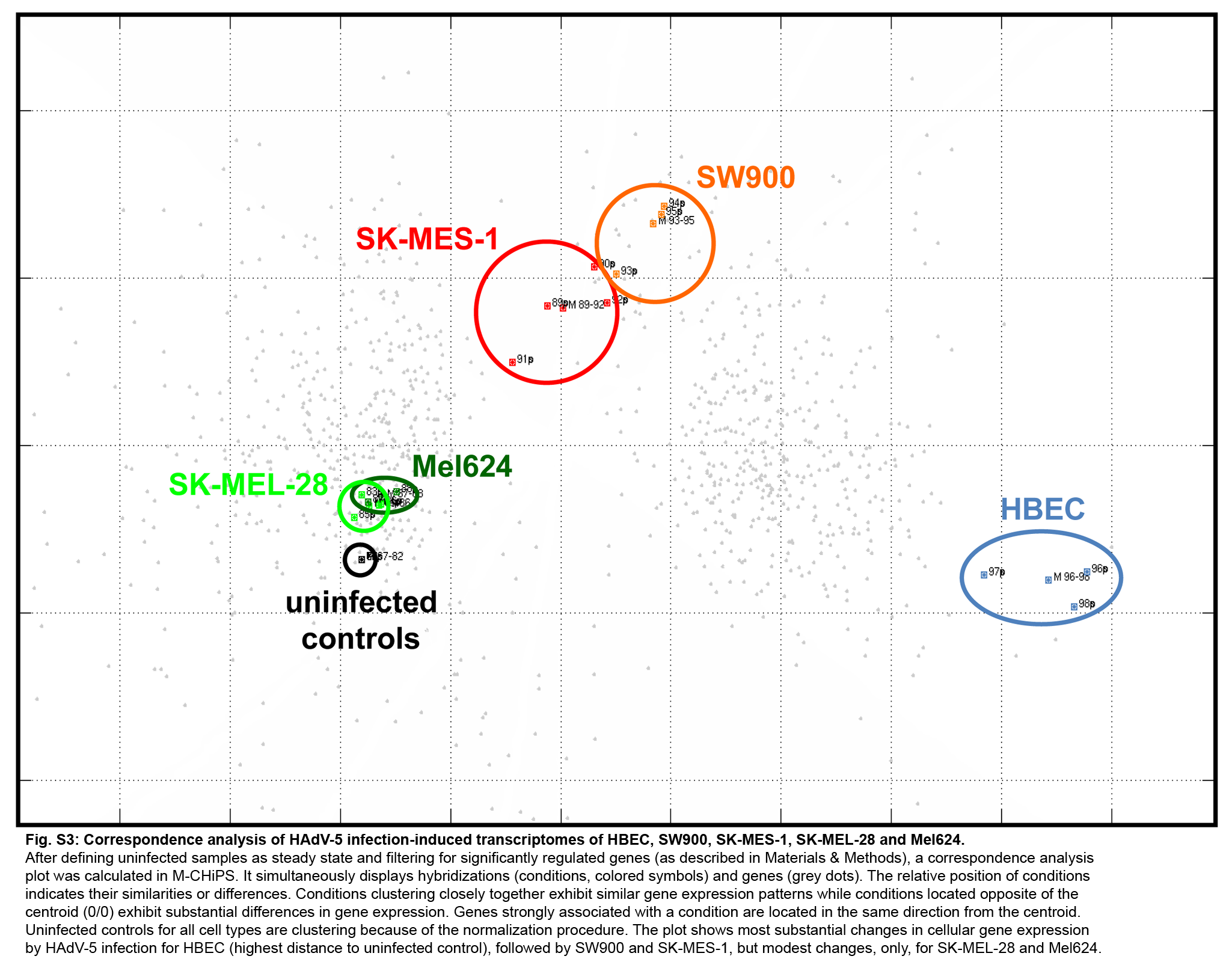

Supplement: Figure S3 — Correspondence analysis of HAdV-5 infection-induced transcriptomes of HBEC, SW900, SK-MES-1, SK-MEL-28 and Mel624. After defining uninfected samples as steady state and filtering for significantly regulated genes (as described in Materials & Methods), a correspondence analysis plot was calculated in M-CHiPS. It simultaneously displays hybridizations (conditions, colored symbols) and genes (grey dots). The relative position of conditions indicates their similarities or differences. Conditions clustering closely together exhibit similar gene expression patterns while conditions located opposite of the centroid (0/0) exhibit substantial differences in gene expression. Genes strongly associated with a condition are located in the same direction from the centroid. Uninfected controls for all cell types are clustering because of the normalization procedure. The plot shows most substantial changes in cellular gene expression by HAdV-5 infection for HBEC (highest distance to uninfected control), followed by SW900 and SK-MES-1, but modest changes, only, for SK-MEL-28 and Mel624. (TIF) [file pone.0027934.s003.tif]

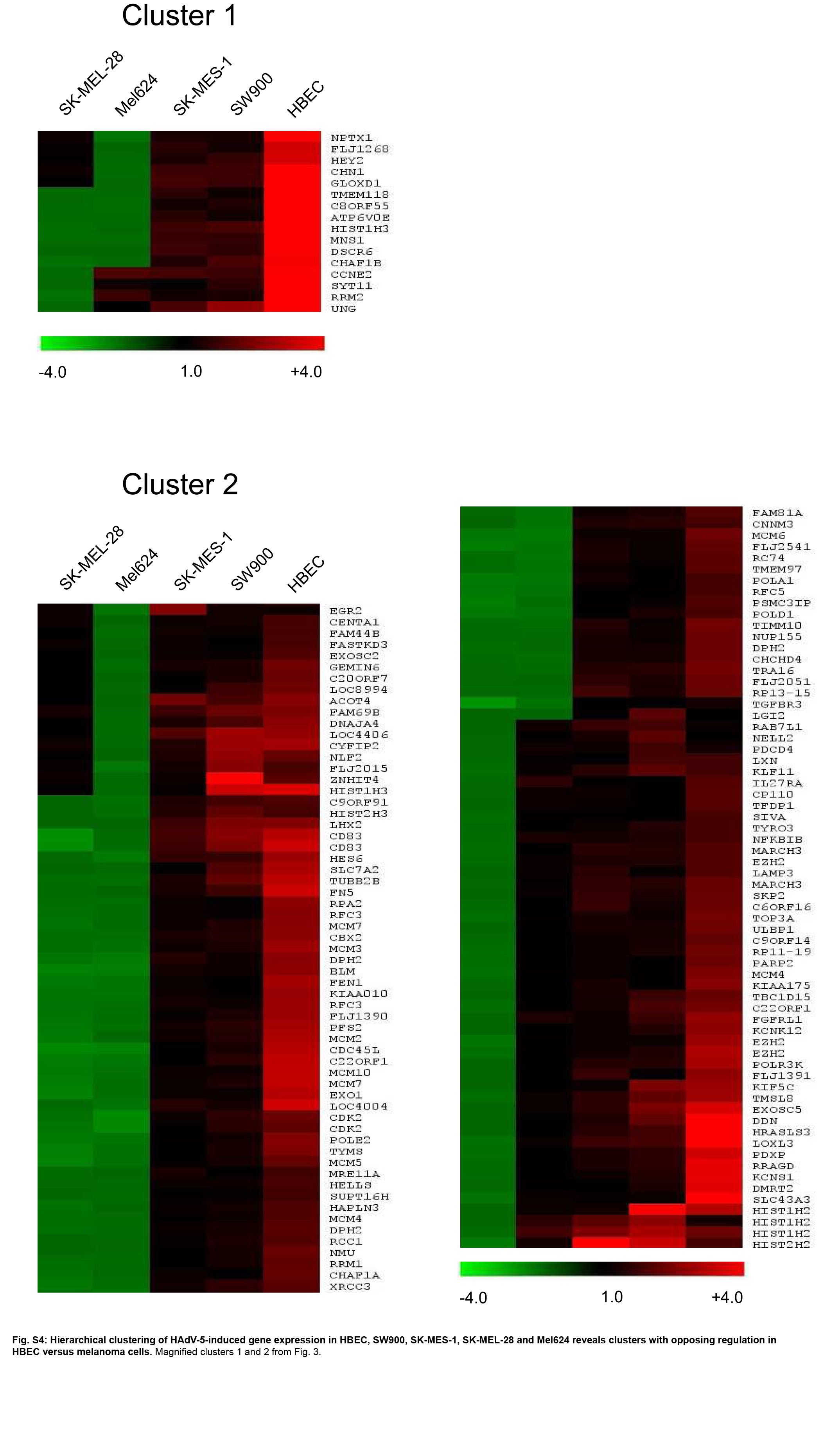

Supplement: Figure S4 — Hierarchical clustering of HAdV-5-induced gene expression in HBEC, SW900, SK-MES-1, SK-MEL-28 and Mel624 reveals clusters with opposing regulation in HBEC versus melanoma cells. Magnified clusters 1 and 2 from Fig. 3. (TIF) [file pone.0027934.s004.tif]

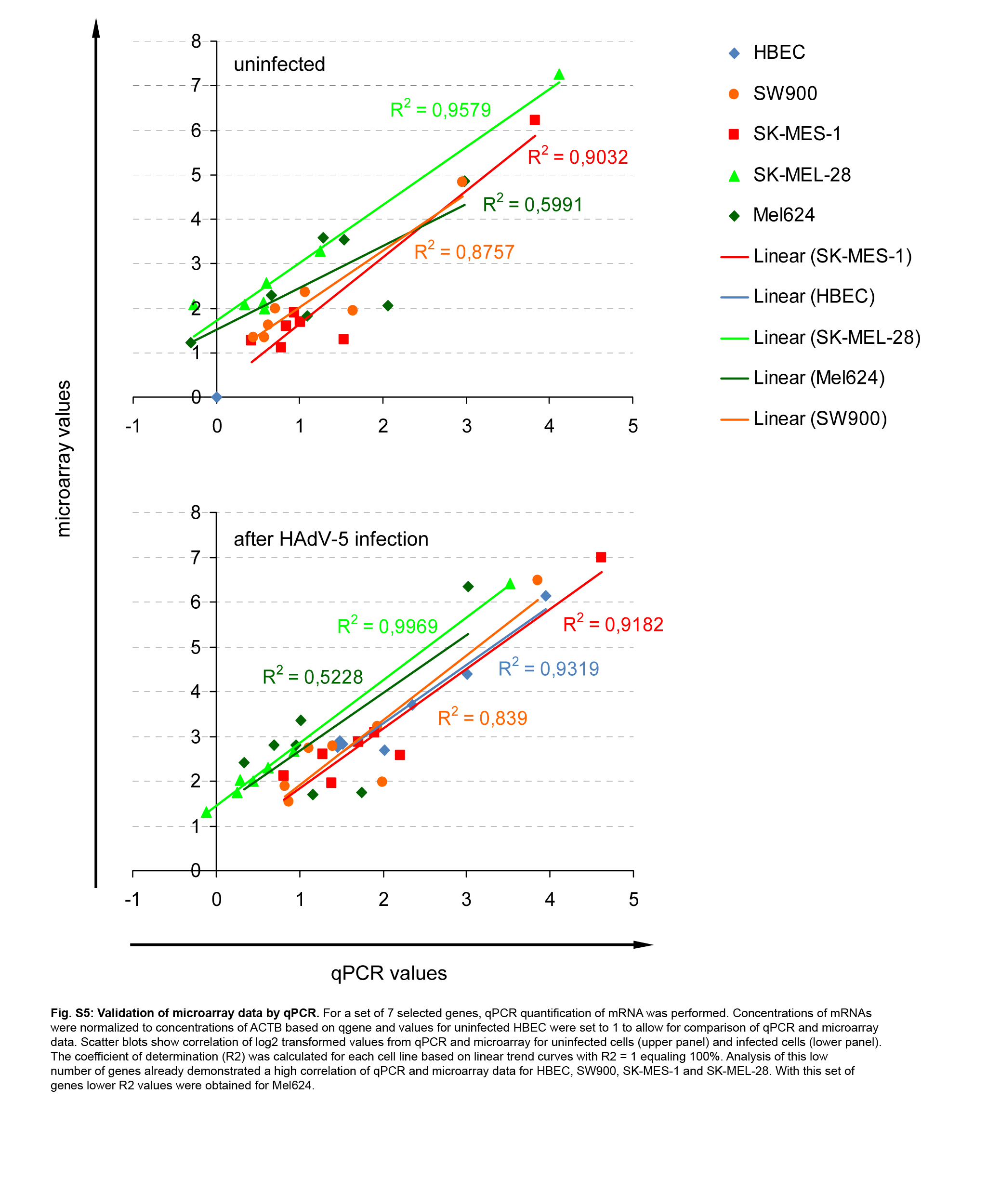

Supplement: Figure S5 — Validation of microarray data by qPCR. For a set of 7 selected genes, qPCR quantification of mRNA was performed. Concentrations of mRNAs were normalized to concentrations of ACTB based on qgene and values for uninfected HBEC were set to 1 to allow for comparison of qPCR and microarray data. Scatter blots show correlation of log2 transformed values from qPCR and microarray for uninfected cells (upper panel) and infected cells (lower panel). The coefficient of determination (R2) was calculated for each cell line based on linear trend curves with R2 = 1 equaling 100%. Analysis of this low number of genes already demonstrated a high correlation of qPCR and microarray data for HBEC, SW900, SK-MES-1 and SK-MEL-28. With this set of genes lower R2 values were obtained for Mel624. (TIF) [file pone.0027934.s005.tif]

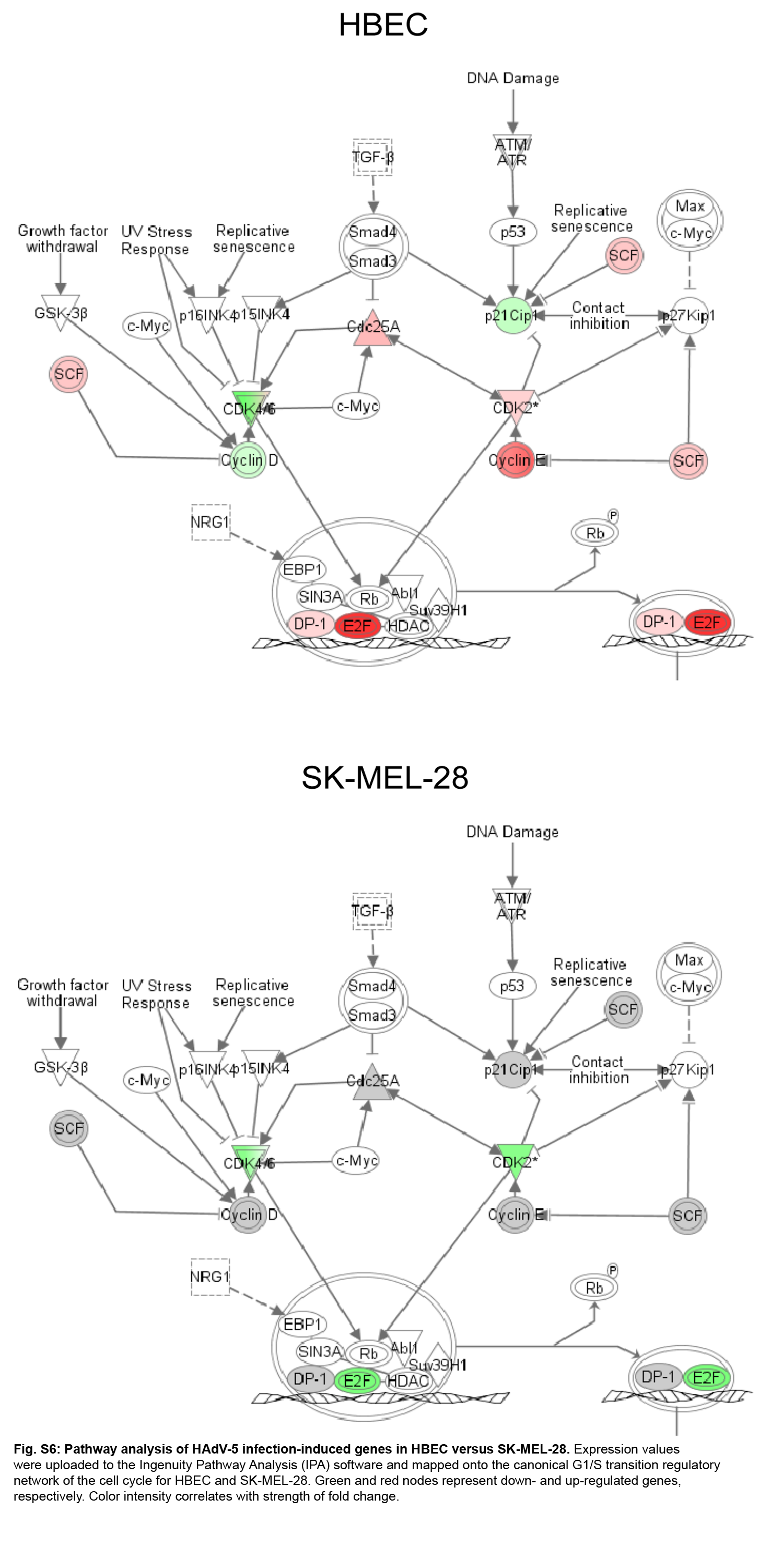

Supplement: Figure S6 — Pathway analysis of HAdV-5 infection-induced genes in HBEC versus SK-MEL-28. Expression values were uploaded to the Ingenuity Pathway Analysis (IPA) software and mapped onto the canonical G1/S transition regulatory network of the cell cycle for HBEC and SK-MEL-28. Green and red nodes represent down- and up-regulated genes, respectively. Color intensity correlates with strength of fold change. (TIF) [file pone.0027934.s006.tif]
