# Supplementary material for: Replication and Virus-Induced Transcriptome of HAdV-5 in Normal Host Cells versus Cancer Cells - Differences of Relevance for Adenoviral Oncolysis
Source: PLoS One. 2011 Nov 30;6(11):e27934. doi: 10.1371/journal.pone.0027934 (PMC3227638; doi:10.1371/journal.pone.0027934)
Supplement: Table S1 — Infectious particle titers used to achieve 80% tranduction efficiency for individual cell types as determined by transduction with HAdV-5 CMV-gfp and quantification of GFP positive cells in the living cell fraction by fluorescence cytometry. (DOC) [file pone.0027934.s007.doc]

**Supporting Information Table S1**

**Table S1**: Infectious particle titers used to achieve 80 % tranduction efficiency for individual cell types as determined by transduction with HAdV-5 CMV-gfp and quantification of GFP positive cells in the living cell fraction by fluorescence cytometry.

| **cell line** | **origin** | **TCID_50_ titer/cell** |
| --- | --- | --- |
| A549 | Lung adenocarcinoma | 500 |
| HBEC, both donors | primary lung epithelium | 800 |
| HFF | primary human fibroblasts | 1500 |
| Mel624 | melanoma | 400 |
| PHK | primary human keratinocytes | 800 |
| SK-MEL-28 | melanoma | 300 |
| SK-MES-1 | lung squamous cell carcinoma | 700 |
| SW900 | lung squamous cell carcinoma | 1500 |
