# Supplementary material for: Replication and Virus-Induced Transcriptome of HAdV-5 in Normal Host Cells versus Cancer Cells - Differences of Relevance for Adenoviral Oncolysis
Source: PLoS One. 2011 Nov 30;6(11):e27934. doi: 10.1371/journal.pone.0027934 (PMC3227638; doi:10.1371/journal.pone.0027934)
Supplement: Table S2 — Gene annotations and genes significantly accumulating in the top 100 down-regulated genes in HBEC. (DOC) [file pone.0027934.s008.doc]

**Supporting Information Table S2**

**Table S2**: Gene annotations and genes significantly accumulating in the top 100 down-regulated genes in HBEC.

**Downregulated GO-Terms**

| **GO-Term/**gene | **P-value**/fold induction |
| --- | --- |
| **regulation of cell proliferation** | **3,7E-8** |
| PTHLH | -16,09 |
| PLAU | -11,95 |
| SERPINE | -10,25 |
| THBS1 | -9,35 |
| CDK6 | -7,4 |
| PTGS2 | -7,07 |
| JAG1 | -7,01 |
| ITGA2 | -6,5 |
| SOX9 | -5,91 |
| TGFBR2 | -5,43 |
| F3 | -4,54 |
| VEGFC | -4,37 |
| TRIB1 | -4,06 |
| CAV2 | -4 |
| JUN | -3,86 |
| CAV1 | -3,71 |
| BTG2 | -3,69 |
| KLF10 | -3,68 |
| IL8 | -3,53 |
| HBEGF | -3,49 |
| ETS1 | -3,48 |
| TXNIP | -3,47 |
| BCL2L1 | -3,46 |
| NRP1 | -3,43 |
| PTPRK | -3,4 |
| **blood vessel morphogenesis** | **3,7E-6** |
| **blood vessel development** | **9,9E-6** |
| **vasculature development** | **1,0E-5** |
| ANGPTL4 | -14,17 |
| PLAU | -11,95 |
| THBS1 | -9,35 |
| JAG1 | -7,01 |
| TGFBR2 | -5,43 |
| ZFP36L1 | -4,72 |
| VEGFC | -4,37 |
| TIPARP | -4,28 |
| JUN | -3,86 |
| RASA1 | -3,74 |
| CAV1 | -3,71 |
| IL8 | -3,53 |
| NRP1 | -3,43 |
| **regulation of myeloid cell differentiation** | **4,4E-6** |
| CDK6 | -7,4 |
| JAG1 | -7,01 |
| HMGB3 | -4,62 |
| HIST1H4 | -4,14 |
| JUN | -3,86 |
| ZFP36 | -3,81 |
| KLF10 | -3,68 |
| ETS1 | -3,48 |
| RUNX1 | -3,4 |
| **negative regulation of cell differentiation** | **1,8E-5** |
| PTHLH | -12,73 |
| CDK6 | -7,4 |
| JAG1 | -7,01 |
| SOX9 | -5,91 |
| FST | -5,77 |
| HMGB3 | -4,62 |
| ABCA1 | -4,45 |
| HIST1H4 | -4,14 |
| ZFP36 | -3,81 |
| CAV1 | -3,71 |
| NRP1 | -3,43 |
| RUNX1 | -3,4 |
| **negative regulation of cell proliferation** | **5,3E-5** |
| PTHLH | -12,73 |
| THBS1 | -9,35 |
| CDK6 | -7,4 |
| PTGS2 | -7,07 |
| TGFBR2 | -5,43 |
| TRIB1 | -4,06 |
| CAV2 | -4 |
| JUN | -3,86 |
| CAV1 | -3,71 |
| BTG2 | -3,69 |
| KLF10 | -3,68 |
| IL8 | -3,53 |
| ETS1 | -3,48 |
| PTPRK | -3,4 |
| **regulation of response to external stimulus** | **9,0E-5** |
| PLAU | -11,95 |
| SERPINE | -10,25 |
| THBS1 | -9,35 |
| PTGS2 | -7,07 |
| ITGA2 | -6,5 |
| TGFBR2 | -5,43 |
| F3 | -4,54 |
| ZFP36 | -3,81 |
| CAV1 | -3,71 |
| IL8 | -3,53 |
| **response to mechanical stimulus** | **1,7E-4** |
| ITGA2 | -6,5 |
| TGFBR2 | -5,43 |
| FOS | -4,65 |
| JUN | -3,86 |
| CAV1 | -3,71 |
| BTG2 | -3,69 |
| TXNIP | -3,47 |
| **regulation of apoptosis** | **1,8E-4** |
| **regulation of cell death** | **1,8E-4** |
| ANGPTL4 | -14,17 |
| PHLDA1 | -10,8 |
| THBS1 | -9,35 |
| PTGS2 | -7,07 |
| SOX9 | -5,91 |
| TNFRSF6 | -5,06 |
| IER3 | -4,68 |
| F3 | -4,54 |
| NET1 | -4,39 |
| JUN | -3,86 |
| RASA1 | -3,74 |
| MCL1 | -3,73 |
| BTG2 | -3,69 |
| KLF10 | -3,68 |
| CEBPB | -3,57 |
| TNFAIP3 | -3,56 |
| ETS1 | -3,48 |
| TXNIP | -3,47 |
| BCL2L1 | -3,46 |
